# Demographic predictors of treatments and surgical complications of lumbar degenerative diseases: An analysis of over 250,000 patients from the National Inpatient Sample

**DOI:** 10.1097/MD.0000000000029065

**Published:** 2022-03-18

**Authors:** Omar M. Al Jammal, Shane Shahrestani, Arash Delavar, Nolan J. Brown, Julian L. Gendreau, Brian V. Lien, Ronald Sahyouni, Luis Daniel Diaz-Aguilar, Omar S. Shalakhti, Martin H. Pham

**Affiliations:** ^a^ *Department of Neurosurgery, University of California San Diego School of Medicine, San Diego, CA,* ^b^ *Keck School of Medicine of the University of Southern California, Los Angeles, CA,* ^c^ *Department of Medical Engineering, California Institute of Technology, Pasadena, CA,* ^d^ *University of California Irvine School of Medicine, Irvine, CA,* ^e^ *Department of Biomedical Engineering, Johns Hopkins Whiting School of Engineering, Baltimore, MD.*

**Keywords:** decompression, degenerative lumbar surgery, disparities, fusion, laminectomy, National Inpatient Sample, postoperative complications, spinal stenosis

## Abstract

This was a national database study.

To examine the role of comorbidities and demographics on inpatient complications in patients with lumbar degenerative conditions.

Degenerative conditions of the lumbar spine account for the most common indication for spine surgery in the elderly population in the United States. Significant studies investigating demographic as predictors of surgical rates and health outcomes for degenerative lumbar conditions are lacking.

Data were obtained from the National Inpatient Sample from 2010 to 2014 and International Classification of Diseases, 9th revision, Clinical Modification codes were used to identify patients with a primary diagnosis of degenerative lumbar condition. Patients were stratified based on demographic variables and comorbidity status. Multivariate regression analyses were used to determine whether any individual demographic variables, such as race, sex, insurance, and hospital status predicted postoperative complications.

A total of 256,859 patients were identified for analysis. The rate of overall complications was found to be 16.1% with a mortality rate of 0.10%. Female, Black, Hispanic, and Asian/Pacific Islander patients had lower odds of receiving surgical treatment compared to White patients (*P*<.001). Medicare and Medicaid patients were less likely to be surgically managed than patients with private insurance (OR = 0.75, 0.37; *P*<.001, respectively). Urban hospitals were more likely to provide surgery when compared to rural hospitals (*P* < .001). Patients undergoing fusion had more complications than decompression alone (*P* < .001). Females, Medicare insurance status, Medicaid insurance status, urban hospital locations, and certain geographical locations were found to predict postoperative complications (*P* < .001).

There were substantial differences in surgical management and postoperative complications among individuals of different sex, races, and insurance status. Further investigation evaluating the effect of demographics in spine surgery is warranted to fully understand their influence on patient complications.

## 1. Introduction

Lumbar degenerative disease is a chronic pathology of the lumbar intervertebral disks or vertebral bodies, often presenting in elderly individuals and exacerbating with advanced age.^[1,2]^ Oftentimes, degenerative disease of the lumbar intervertebral disks occurs due to reduced water content, collagen distribution, and proteoglycan concentration. The resulting degeneration may be visualized as hypointense signals on T2-weighted magnetic resonance imaging.^[3]^ Disruption of the architecture of lumbar intervertebral disks following degenerative changes poses a high risk for disk herniation, which may impinge on adjacent nerve roots leading to neuropathy and pain. Roughly 90% of lumbar disk herniations and nerve compressions occur between L4-L5 and L5-S1, which may result in radiculopathy in the areas of corresponding dermatomes in the lower limbs.^[4,5]^

Although age-related degenerative diseases of the lumbar spine are well documented in the literature,^[6-8]^ demographic predictors of poor patient outcomes, including race, insurance type, sex, and hospital status, have yet to be thoroughly evaluated. This study utilizes 5 years of a national United States administrative hospital database to query all patients diagnosed with lumbar degenerative disease, their relevant demographics, and their inpatient complication and management profiles. Through a set of multivariate analyses, the authors aim to identify demographic predictors of poor patient outcomes within the patient cohort. Such an understanding would undoubtedly aid in patient triage and risk stratification prior to surgical intervention and assist surgeons in developing proper follow up strategies depending on patient demographics and risks.

## 2. Materials and methods

Data were obtained from the National Inpatient Sample (NIS), a Healthcare Cost and Utilization Project database which includes a 20% sample of discharges from Healthcare Cost and Utilization Project-participating hospitals; this amounts to over 7 million discharges per year. This study was exempt from institutional review board approval due to the de-identified nature of this database. This study includes data from 2010 to 2014. International Classification of Diseases, 9th revision, Clinical Modification codes were used to identify all patients in the NIS with a primary diagnosis of a degenerative condition of the lumbar spine between 2010 and 2014. This includes diagnoses of lumbar or lumbosacral intervertebral disc disorder with or without myelopathy (722.1, 722.52, 722.73), spondylolysis of the lumbosacral region (756.11), lumbago (724.6), and other unspecified disc disorders of the lumbar region (724.2). A total of 256,859 patients were identified and were examined for 1 of 3 surgical outcomes: decompression alone (3, 3.09, 80.5, 80.51), simple fusion involving 3 or less vertebral levels (81, 81.04, 81.05, 81.06, 81.07, 81.08, 81.62), and complex fusion involving greater than 3 vertebral levels or a 360 degree spinal fusion (81.61, 81.63, 81.64).

The patients were stratified by different demographic variables including age, sex, race, primary insurance, hospital teaching status, and geographic region. In addition, patients were stratified both by comorbidity status using the Charlson Comorbidity Index (CCI) as well as individual diagnoses such as spinal deformity (scoliosis, kyphosis, and lordosis), congestive heart failure, cardiac arrhythmias, pulmonary circulatory disorders, hypertension, paralysis, neurological disorders, renal failure, liver disease, coagulopathy, and fluid and electrolyte disorders. Surgical patients were assessed for various complications including implant-related complications, wound-related complications, incidental durotomy, laceration or puncture, hemorrhage, bacteremia, postoperative infection, postoperative shock, myocardial infarction, iatrogenic stroke, neurologic complications, venous thromboembolism, urinary complications, and death.

A series of multivariate Poisson regression analyses was used to determine if any individual demographic variable predicted a surgical outcome such as the odds of receiving any surgery (decompression or fusion) or the odds of receiving a fusion in particular. Furthermore, a series of regressions was used to determine if any individual demographic variable as well as any particular type of operation (decompression, simple fusion, or complex fusion) predicted surgical complications, including the need for a revision operation. Lastly, multivariate regressions were used to assess how comorbidity status, including both Charlson indices and individual diagnoses, predicted surgical mortality. All multivariate regression models were controlled for age, sex, primary insurance type, median household income, geographic region, hospital teaching status, comorbidity status, and additional variables displayed in the Results section tables. Multivariate analyses were presented as odds ratios, with corresponding 95% confidence intervals and *P* values; [Odds Ratio (OR) (95% confidence interval), *P*<x]. Given the cohort sample size and the series of multivariate analyses, a *P* value < .05 was used to determine significance. Data extraction, analyses, and statistical tests were done with Stata version 11.2 (StataCorp, College Station, TX) and RStudio version 3.5.1 (R Core Team, Vienna, Austria).

## 3. Results

### 3.1. Demographics

A total of 256,859 patients were identified for analysis. Within this cohort, 221,407 (86.2%) received surgical intervention when analyzing all hospital locations. Of these, 35.7% were decompression operations, 45.1% were simple lumbar fusion operations, and 5.4% were complex fusion operations. The average age within the cohort was 58.2 ± 15.6 years with 51.0% being female and an average CCI of 0.62 ± 1.02. With respect to race, 81.6% of patients were White, 7.7% of patients were Black, 6.5% of patients were Hispanic, and 1.1% of patients were Asian/Pacific Islander (Table S1, Supplemental Digital Content, http://links.lww.com/MD/G654). Within the entire cohort, 41.2% of patients had private insurance, 41.3% had Medicare, and 6.1% had Medicaid. A minority of patients (5.5%) were admitted to rural hospitals, while 43.2% of patients were admitted to urban non-teaching hospitals and 51.5% of patients were admitted to urban teaching hospitals. Similarly, the hospital geographic distribution included 19.4% in the Northeast, 41.5% in the South, 19.2% in the Midwest, and 19.7% in the West. Lastly, the average mortality rate within all patients included in this study was found to be 0.10% (Table 1).

**Table 1:** Characteristics of patients with a primary degenerative condition of the lumbar spine from 2010 to 2014 in the National Inpatient Sample*.

**Year**		**2010**	**2011**	**2012**	**2013**	**2014**
Total patients		57,786	57,645	50,519	46,201	44,708
Total operations (%)		49,417 (85.5)	49,813 (86.4)	43,553 (86.2)	40,120 (86.8)	38,504 (86.1)
	% decompression	37.0	38.0	36.8	34.5	31.3
	% simple fusion	43.3	43.3	44.3	46.5	49.2
	% complex fusion	5.3	5.1	5.1	5.8	5.9
Female (%)		51.5	51.0	51.1	50.8	50.5
Mean age (SD*)		58 (15.9)	58 (15.7)	57.9 (15.7)	58.4 (15.5)	58.9 (15.2)
Age categories (%)	
	Age: 1-17	0.3	0.2	0.3	0.3	0.3
	Age: 18-44	21.4	21.1	21.1	19.8	18.6
	Age: 45-64	40.8	41.0	41.0	41.1	40.9
	Age: 65-84	34.3	34.5	34.5	36.0	37.3
	Age: 85+	3.2	3.1	3.1	2.9	3.0
Race	
	White (%)	82.9	81.7	81.3	81	80.5
	Black (%)	7.7	7.5	7.6	7.9	8.1
Hispanic (%)	5.4	7.2	6.5	6.7	6.9
	Asian/Pacific Islander (%)	1.0	1.0	1.2	1.2	1.4
	Other (%)	2.5	2.3	3	2.7	2.7
Primary insurance	
	Private (%)	42.6	42	41.3	40.2	39.3
	Medicare (%)	40.1	40.6	40.9	42.2	43.2
	Medicaid (%)	5.6	5.4	6.2	6.0	7.5
Hospital teaching status	
	Rural	8.3	4.6	5.2	5.0	4.0
	Urban, non-teaching	50.0	47.4	43.4	42.6	29.4
	Urban, teaching	42.1	48	51.4	52.4	67.6
Geographic region	
	Northeast (%)	18.4	18.5	20.1	20.3	20.2
	South (%)	45.1	43.4	38.4	39.5	39.7
	Midwest (%)	17.7	17.3	21.0	20.5	20.3
	West (%)	18.8	20.1	20.4	19.8	19.7
Death (%)		0.11	0.09	0.11	0.12	0.09
Mean Charlson Comorbidity Index score (SD)		0.59 (0.99)	0.59 (0.99)	0.61 (1.02)	0.64 (1.06)	0.66 (1.07)
SD = standard deviation.						

^*^Unweighted data, national estimates not provided.

### 3.2. Surgical vs conservative management

After adjusting for age, CCI, and median household income, female patients had lower odds of receiving surgery than male patients (OR = 0.79; 95% CI = 0.77-0.81; *P*<.001) as shown in Table S2, Supplemental Digital Content, http://links.lww.com/MD/G655. In addition, Black patients (OR = 0.51; 95% CI = 0.49-0.53; *P*<.001), Hispanic patients (OR = 0.53; 95% CI = 0.50-0.55; *P*<.001), and Asian/Pacific Islander patients (OR = 0.77; 95% CI = 0.69-0.85; *P*<.001) all had lower odds of receiving surgical treatment compared to white patients. Similar trends were also found with regards to insurance type, with Medicare (OR = 0.75; 95% CI = 0.72-0.77; *P*<.001) and Medicaid (OR = 0.37; 95% CI = 0.35-0.38; *P*<.001) patients receiving surgical treatment at a significantly lower rate compared to patients with private insurance. Lastly, patients treated at urban non-teaching (OR = 1.76; 95% CI = 1.68-1.85; *P*<.001) and urban teaching (OR = 2.45; 95% CI = 2.34-2.57; *P*<.001) hospitals were found to have higher odds of receiving surgery compared to patients at rural hospitals, and patients admitted to hospitals in the Midwest (OR = 1.21; 95% CI = 1.17-1.25; *P*<.001), South (OR = 1.77; 95% CI = 1.71-1.83; *P* <.001), and West (OR = 1.75; 95% CI = 1.68-1.81; *P*<.001) had a higher odds of receiving surgical treatment compared to those treated in the Northeast (Table 2). Several variables were also associated with receiving fusion specifically in contrast to decompression (Table S3, Supplemental Digital Content, http://links.lww.com/MD/G656).

**Table 2:** **Odds ratio for receiving surgery in patients with a primary degenerative condition of the lumbar spine** - multivariable Poisson regression, NIS data, 2010 to 2014*.

**Demographic**		**Odds ratio**	**95% confidence interval**	***P***
Gender	
	Male	Reference
	Female	0.79	0.77, 0.81	*P* < .001
Race	
	White	Reference
	Black	0.51	0.49, 0.53	*P*<.001
	Hispanic	0.53	0.50, 0.55	*P*<.001
	Asian/Pacific Islander	0.77	0.69, 0.85	*P* < .001
	Other	0.88	0.82, 0.95	*P* = .001
Primary insurance	
	Private	Reference
	Medicare	0.75	0.72, 0.77	*P* < .001
	Medicaid	0.37	0.35, 0.38	*P* < .001
Hospital teaching status	
	Rural	Reference
	Urban, non-teaching	1.76	1.68, 1.85	*P* < .001
	Urban, teaching	2.45	2.34, 2.57	*P*<.001
Geographic region	
	Northeast	Reference
	Midwest	1.21	1.17, 1.25	*P*<.001
	South	1.77	1.71, 1.83	*P*<.001
	West	1.75	1.68, 1.81	*P* < .001
Year	
	2010	Reference
	2011	1.05	1.01, 1.08	*P* = .013
	2012	1.07	1.03, 1.10	*P*<.001
	2013	1.15	1.10, 1.19	*P*<.001
	2014	1.04	0.98, 1.08	*P* = .07
Spinal deformity	
	None	Reference
	Scoliosis, kyphosis, lordosis	1.92	1.80, 2.04	*P*<.001
NIS = National Inpatient Sample.				

^*^Also adjusted for age, Charlson comorbidity status, and median household income.

### 3.3. Complications in patients treated with surgery

Within all patients, 1.1% had implant-related complications, 0.2% had wound-related complications, 4.2% reported incidental durotomy, 0.3% reported laceration of a non-target structure, 8.6% experienced hemorrhage, hematoma, or seroma, 0.2% had septicemia, 0.1% experienced postoperative infection, 0.02% experienced postoperative shock, 0.2% experienced myocardial infarction, 0.03% experienced stroke, 0.5% reported neurologic complications, 0.3% experienced venous thromboembolism, 2.7% developed urinary complications, and 0.08% died. The total complication rate, including any of the aforementioned complications, was 16.1% within all patients (Table 3).

**Table 3:** Complication rates in surgical patients with a primary degenerative condition of the lumbar spine from 2010 to 2014 in the National Inpatient Sample*.

**Year**	**2010**	**2011**	**2012**	**2013**	**2014**
Total patients (N)	49,417	49,813	43,553	40,120	38,504
Complication rates
Total complication rate* (%)	15.7	16.6	15.7	16.2	16.4
% implant-related complication	1.09	1.07	1.02	1.13	1.11
% wound-related complication	0.22	0.20	0.20	0.17	0.18
% incidental durotomy	4.19	4.28	4.11	4.14	4.20
% Laceration/puncture (vessel, nerve, organ)	0.46	0.33	0.31	0.23	0.28
% hemorrhage/hematoma/seroma	7.89	9.11	8.48	8.73	9.04
% bacteremia/septicemia	0.17	0.22	0.19	0.21	0.19
% postoperative infection	0.17	0.16	0.14	0.13	0.12
% postoperative shock	0.06	0.05	0.00	0.00	0.00
% myocardial infarction	0.20	0.22	0.15	0.17	0.18
% iatrogenic stroke	0.04	0.05	0.04	0.03	0.00
% neurologic complication	0.58	0.56	0.50	0.54	0.46
% venous thromboembolism*	0.33	0.33	0.38	0.34	0.35
% urinary complication**	2.80	2.79	2.53	2.55	2.52
Death (%)	0.08	0.07	0.08	0.09	0.07

^*^Total complication rate excluding death. Venous thromboembolism includes diagnostic codes for pulmonary embolism and thromboembolism in deep vessels of the lower extremities.

^**^Urinary complication includes diagnostic codes for UTI and unspecified urinary complication. UTI = Urinary Tract Infection.

Models capable of predicting whether patients developed 1 or more complications showed that patients who received simple (OR = 1.74; 95% CI = 1.70-1.79; *P*<.001) or complex (OR = 4.08; 95% CI = 3.91-4.25; *P*<.001) lumbar fusion had higher odds of developing a complication compared to those who received decompression surgery. Although females were found to have a higher odd of experiencing acute postsurgical complications (OR = 1.33; 95% CI = 1.30-1.36; *P*<.001) compared to males, no significant difference in complication rates was found across patients of different racial groups. Furthermore, insurance status (*P*<.001), hospital teaching status (*P*<.03), and geography (*P*<.001) were found to significantly predict postoperative complication rates (Table 4).

**Table 4:** Odds ratio for surgical complication* **in patients with a primary degenerative lumbar condition** - multivariable Poisson regression, NIS data, 2010 to 2014*.

**Demographic**		**Odds Ratio**	**95% Confidence Interval**	***P***
Surgery type	
	Decompression	Reference
	Simple fusion (2-3 levels)	1.74	1.70, 1.79	*P* < .001
	Complex fusion (4+ levels)	4.08	3.91, 4.25	*P* < .001
Gender	
	Male	Reference
	Female	1.33	1.30, 1.36	*P* < .001
Race	
	White	Reference
	Black	1.15	1.10, 1.21	*P*<.001
	Hispanic	0.97	0.97, 1.02	*P* = .236
	Asian/Pacific Islander	1.04	0.93, 1.16	*P* = .493
	Other	1.05	0.97, 1.07	*P* = .227
Primary insurance	
	Private	Reference
	Medicare	1.11	1.07, 1.14	*P*<.001
	Medicaid	1.15	1.08, 1.21	*P*<.001
Hospital teaching status	
	Rural	Reference
	Urban, non-teaching	1.07	1.01, 1.13	*P* = .027
	Urban, teaching	1.32	1.24, 1.40	*P* < .001
Geographic region	
	Northeast	Reference
	Midwest	1.19	1.14, 1.24	*P*<.001
	South	1.07	1.03, 1.11	*P*<.001
	West	1.20	1.16, 1.25	*P*<.001
Spinal deformity	
	None	Reference
	Scoliosis, kyphosis, lordosis	1.43	1.37, 1.49	*P* < .001
Also adjusted for age, Charlson comorbidity status, and median household income.				
NIS = National Inpatient Sample; HTN = Hypertension.				

^*^One or more surgical complications listed in Table 3, excluding death.

Lastly, models were developed to predict mortality in patients surgically treated for lumbar degenerative disease. Patients treated with simple and complex fusion procedures were found to die at a higher rate compared to those treated with decompression surgery (*P*<.001). Patient comorbidities and CCI scores were also found to independently predict mortality within patients receiving surgical treatment (Table 5).

**Table 5:** **Odds ratio of increasing mortality in surgical patients with a primary degenerative condition of the lumbar spine** - multivariable Poisson regression, NIS data, 2010 to 2014*.

**Demographic**		**Odds Ratio**	**95% Confidence Interval**	***P***
Surgery type	
	Decompression	Reference
	Simple fusion (2-3 levels)	2.07	1.42, 3.07	*P*<.001
	Complex Fusion (4+ levels)	2.75	1.65, 4.55	*P*<.001
Comorbidity	
	Congestive heart failure	2.79	1.77, 4.30	*P*<.001
	Cardiac arrhythmias	2.90	2.03, 4.10	*P*<.001
	Pulmonary circulatory disorder	9.21	5.33, 15.46	*P*<.001
	HTN, uncomplicated	0.59	0.41, 0.83	*P* = .003
	Paralysis	2.86	1.54, 4.95	*P*<.001
	Other neurological disorder	6.34	4.31, 9.15	*P*<.001
	Renal failure	3.70	1.71, 7.40	*P*<.001
	Liver disease	7.99	4.58, 13.31	*P*<.001
	Coagulopathy	3.41	2.15, 5.26	*P*<.001
	Fluid & electrolyte disorder	6.02	4.31, 8.41	*P* < .001
Comorbidity status	
	Charlson Score 0	Reference
	Charlson Score 1	2.29	1.57, 3.34	*P*<.001
	Charlson Score 2	2.26	1.31, 3.75	*P* = .002
	Charlson Score 3	5.98	3.40, 10.11	*P*<.001
	Charlson Score 4	11.89	6.36, 21.00	*P*<.001
	Charlson Score 5	19.08	8.23, 38.87	*P*<.001
	Charlson Score 6 or greater	7.67	1.24, 25.07	*P* = .005
HTN = Hypertension, NIS = National Inpatient Sample.				

^*^Also adjusted for age, insurance type, race, median household income, hospital region, and coexisting spinal deformity.

## 4. Discussion

In this study, the authors conducted a 5-year retrospective analysis of demographic predictors of patient management and complications in a large sample of patients diagnosed with lumbar degenerative disease. Multivariate predictive modelling allowed the study to control for patient-specific confounding variables, and the findings suggest that sex, race, insurance status, hospital type, and geography may influence whether patients receive surgical vs conservative treatment and develop postoperative complications. Furthermore, additional multivariate models were created to evaluate patient factors that predict mortality which found that procedure type, individual medical comorbidities, and CCI scoring may accurately predict inpatient mortality within our cohort.

Overall during the timeframe of this study, it appears that rate of inpatient stays was decreasing from 2010 to 2014 (57,786 and 44,708 patients, respectively). This could potentially be the result of an increasing number of patients being treated through more conservative means such as spinal cord stimulators, epidural injections, or other forms of non-operative treatment.^[9,10]^ In addition, the advent of outpatient spinal surgery for degenerative diseases such as the increasing use of outpatient decompression and fusion surgeries could also contribute to this decreasing rate of inpatient stays.^[11]^

Prior studies have demonstrated that patient demographics are associated with postoperative outcomes in a variety of spinal procedures. A study conducted by Triebel et al^[12]^ in 2017 found that Swedish women who received lumbar fusion surgery for degenerative disk disease had outcomes comparable to those of men, and showed no significant difference in quality of life and return to work with 2 years of follow up. Conversely, Kim et al^[13]^ reported that females may receive surgical treatment of lumbar degenerative disease less frequently than males when patient management is approached with a preference-based, shared decision-making process, similar to the findings described in this paper. This hypothesis is further bolstered by a recent systematic review by MacLean et al^[14]^ which explains that females have worse absolute pain, quality of life, and disability following surgical treatment of lumbar degenerative disease compared to males. As such, conflicting hypotheses in the literature make it difficult to ascertain the role of sex on patient outcomes in those with lumbar degenerative disease. To the authors’ knowledge, this study is the largest study that evaluates the role of demographics in predicting patient outcomes within the context of lumbar degenerative disease. Such large patient numbers increase the power of the study and help establish the predictive capabilities of demographics in patient care.

Additionally, there currently exists a limited and conflicting body of literature evaluating race in the context of spinal pathology and surgery. The Spine Patient Outcomes Research Trial found that White patients were significantly more likely to receive surgical treatment of spinal pathologies compared to Black patients, and this finding is further supported by the results of this multivariate analysis.^[15]^ Similar trends, in which Black patients have a higher rate of morbidity and mortality compared to patients of other races, have also been demonstrated within anterior cervical spine surgery, spinal fusion procedures, and spinal cord tumor resection procedures.^[15-18]^ However, similar analyses of race as a risk factor in spine surgery is limited for patients of Hispanic and Asian/Pacific Islander races. In this study, the authors have a large enough patient cohort to develop multivariate models for patients who identify with several racial groups, with corresponding odds ratios and risk calculations.

Additionally, patient insurance type, hospital teaching status, and geography was found to be associated with treatments and outcomes. Over the timeframe of this database analysis, there was a decreasing rate of patients receiving treatment at rural and urban non-teaching status while an increasing rate of treatment was found in hospitals with urban teaching status. This could be the result of more patients preferring to be treated by specialists located in large academic centers over time. It could also be that patient in more rural environments, or patients with less severe disease opted to be treated in outpatient centers. Nevertheless, it comes as no surprise that large urban teaching institutions operate more frequently on older patients with additional comorbidities than small rural hospitals. As a result, they often encounter higher rates of postoperative complications.^[19,20]^ Comparably, patients with private insurance have a broader set of options with regards to hospital reputation and geography prior to receiving elective surgery and may even receive spine surgery sooner than patients with Medicare or Medicaid.^[21-23]^ As a result, these patients may exhibit better postoperative outcomes and receive surgical treatment more frequently than their counterparts.

No doubt, demographic-based health disparities in lumbar degenerative disease originate from a wide array of contributing factors. First, implicit biases within physician populations may influence the rate at which surgical treatments are afforded to minority populations.^[24,25]^ Second, variations in access to care, including financial, educational, and geographic barriers, may influence the rate at which certain populations seek medical care and the severity of their conditions at the time of elective surgery.^[26-31]^ Lastly, differences in attitudes toward surgical care among those of different racial or ethnic groups may influence rates of surgical treatment for lumbar degenerative diseases.^[32]^

It is interesting to note that the number of patients within the ages of 1 to 17 ranged from 0.2% to 0.3% across all years of the study as having degenerative disease. Most pediatric spinal surgery is performed due to deformity such as scoliosis or kyphosis. In addition, trauma and cancer are also prominent causes while surgery for degenerative changes is less reported.^[33]^ Indeed, a previous database analysis of 12,000 found approximately a 0.2% rate of lumbar disc diseases in pediatric patients.^[34]^ In addition, 1 retrospective series had patients undergoing fusion for reported degenerative changes that they suspected were due to structural malformation of the spine.^[35]^ Nevertheless, pediatric degenerative changes appear to be not as common as in adults which is further evidenced by this study. Finally, fusion was associated with increased mortality when compared with compression only. Previous retrospective studies and randomized controlled trials directly comparing decompression with fusion surgeries do not appreciate any differences in mortality profiles. However, these studies have low sample sizes <100.^[36-38]^ One hypothesis for this increased in mortality with fusion is that patients requiring these surgeries were unhealthier overall, and thus had increased mortality when compared to decompression patients. Indications for fusion include traumatic causes, infection, tumor, and spinal disease causing instability, which may not be present in patients undergoing only decompression.^[39]^

### 4.1. Limitations

This study has many limitations. This study has the inherent limitations of a retrospective cohort analysis. Namely, the conclusions drawn in this paper are subject to the quantity and quality of patient records included in the NIS database. The data used for analysis come from a narrow window of time and includes data from 2010 to 2014. However, this time period was selected to reduce the risk of confounding due to the mandatory transition to ICD-10 coding in 2015. Given the quality of patient records in the database it is hard to ascertain how many of these surgeries were strongly indicated - a decision to undergo surgery is subject to the individual variation of both patient and provider. Many patient variables that could influence whether or not a patient has surgery, in addition to complications after surgery, were unable to be retrieved and taken into consideration in this analysis. These include whether the patient had minimally invasive surgery, previous treatments, whether the patient had osteoporosis and also patient body mass index. Total case volume of both the hospital and surgeon were also variables that were unable to be accounted for in this study. The indication for surgical treatment likely was also heterogenous and varied from surgeon to surgeon. The NIS transitioned to a different sampling strategy in 2012 and was thus made-up of different contributing hospitals. This could have contributed to some of the year-to-year differences in rates; our study thus did not specifically analyze changes in surgical management our complications across time.

Indeed, this database also only included data of inpatient hospital stays only, thus much of the data of patients treated conservatively without treatment or those who had decompression surgery on an outpatient basis were not retrieved. This could introduce selection bias for patients with more severe disease likely increasing the amount of patients undergoing surgery in our sample compared to the general population.

## 5. Conclusion

Lumbar degenerative disease continues to affect a significant proportion of individuals in advanced ages, and modern treatment strategies involve surgery and conservative management. Demographics, including sex, race, geography, hospital teaching status, and insurance type are associated with how patient lumbar pathologies are managed and frequency of postoperative complications. This knowledge can be used in preoperative counseling of patients who are deciding on surgery and the associated risks. Additionally, a deeper understanding of how surgical management and treatment outcomes differ based on demographics can better inform health policy decisions. This knowledge can inform health policies which educate surgeons and establish more equity across different demographic groups. Further longitudinal research is necessary to fully understand the influence of patient demographics on surgical management and postoperative complication rates in patients diagnosed with lumbar degenerative disease.

## Author contributions

**Conceptualization:** Omar M. Al Jammal, Shane Shahrestani, Martin H. Pham.

**Data curation:** Arash Delavar, Nolan J. Brown.

**Formal analysis:** Omar M. Al Jammal.

**Investigation:** Brian V. Lien.

**Methodology:** Shane Shahrestani.

**Project administration:** Julian Gendreau, Martin H. Pham.

**Supervision:** Martin H. Pham.

**Validation:** Martin H. Pham.

**Writing** - **original draft:** Omar M. Al Jammal.

**Writing** - **review & editing:** Shane Shahrestani, Arash Delavar, Nolan J. Brown, Julian Gendreau, Brian V. Lien, Ronald Sahyouni, Luis Daniel Diaz-Aguilar, Omar S. Shalakhti, Martin H. Pham.
